# A Challenging Case of Idiopathic Hypereosinophilic Syndrome: An Itching in the Brain

**DOI:** 10.7759/cureus.67278

**Published:** 2024-08-20

**Authors:** Amol Dube, Sunita Kumbhalkar, Ishan Verma, Priyadip Maiti, Keshao B Nagpure, Gunjan K Ghodeshwar

**Affiliations:** 1 Department of General Medicine, All India Institute of Medical Sciences, Nagpur, Nagpur, IND; 2 Department of Cardiology, All India Institute of Medical Sciences, Nagpur, Nagpur, IND

**Keywords:** dermatopathic lymphadenitis, orthokeratotic hyperkeratosis, pdgfr, clonal, gleich, imatinib, hypereosinophilia syndrome, idiopathic

## Abstract

Hypereosinophilia (HE) has various causes and treatment remains a challenge when there is no relief to symptoms and a decrease in the eosinophil count. Such cases need extensive laboratory support, but the cause may remain obscured in some cases. This is a case of a 58-year-old known diabetic and hypothyroid female who initially presented with fever secondary to pyelonephritis and later developed severe itching and extensive skin hyperpigmented lesions. The laboratory findings were a persistently elevated eosinophil count and generalized itching that was refractory to treatment. The presentation of episodes of itching was like Gleich syndrome without angioedema and needed an injection of hydrocortisone and chlorpheniramine maleate to treat. Diethylcarbamazine, hydroxyurea, and steroids failed to decrease the eosinophilia as well as the episodic itching. We conducted an extensive workup for mutation studies. The bone marrow eosinophil count was above 20%. Considering it as idiopathic non-steroid-responding HE, imatinib was started, and the patient immediately responded, and the eosinophil count came within the normal range within one month. She has been followed up and closely monitored for the past 1 to 1.5 years with no relapse of symptoms and no rise in the eosinophil count.

## Introduction

Eosinophils are granulocytes containing eosinophilic granules in the cytoplasm that help in combating multicellular parasites in vertebrates. Eosinophils play an important role in inflammation and allergic reactions. The eosinophil count may be increased as a reactive response to allergens or parasites or because of clonal proliferation. High levels of eosinophils may infiltrate tissue and may lead to organ dysfunction. Eosinophils can infiltrate any body tissue, such as the skin, lungs, gastrointestinal tract, heart, nervous system, bone marrow, and joints. It may also lead to thromboembolic events. Hypereosinophilic syndrome (HES) encompasses a range of uncommon conditions characterized by a sustained elevation of eosinophils in the bloodstream (>1.5 x 10^9/L), the absence of identifiable secondary causes for this elevation, and evidence of eosinophil-associated end-organ damage [1]. Tissue HE or eosinophil-associated end-organ damage is identified when (a) eosinophils constitute more than 20% of all nucleated cells in bone marrow sections, (b) a pathologist determines that tissue infiltration by eosinophils is significantly greater than normal physiological levels compared to other inflammatory cells, or (c) staining specifically targeting a known eosinophil granule of protein (e.g., major basic protein) reveals substantial extracellular deposition of the eosinophil-derived proteins, indicating local eosinophil activation. HES may be primary (neoplastic), secondary (reactive), or idiopathic [2]. Clonal eosinophilia, a blood condition, is characterized by the molecular changes causing it. Research so far has pinpointed mutations in genes associated with tyrosine kinases and their signaling roles, such as BCR-ABL, PDGFRA, PDGFRB, and c-KIT. The initial treatment approach for every instance of HES involves administering high doses of steroids. If the tyrosine kinase pathway is involved, it responds to imatinib. But in some cases, though there is no detectable tyrosine kinase pathway signaling defect, it responds dramatically with imatinib. This may be because of a lack of molecular assays that can identify such defects. We present such a case where steroids were not effective and molecular assays were normal, but it dramatically responded to imatinib.

## Case presentation

A 58-year-old female patient, a known case of hypothyroidism and type 2 diabetes mellitus, presented as a case of bilateral pyelonephritis for 15 days. Clinical evaluation revealed a pulse of 112/min and a blood pressure of 94/44 mmHg, suggesting septic shock. There was also a history of intractable itching with erythematous scaly patches for eight days. The examination was suggestive of pallor, conjunctival congestion, and bilateral coarse crackles on the chest auscultation. She was taking tablets of metformin 500 mg twice daily and a tablet of thyroxine 25 mcg daily for coexisting illnesses. She was examined with 2D echocardiography, urine routine microscopy, chest X-ray, and blood culture which were normal. Treatment was started with injection meropenem, levofloxacin, and noradrenaline infusion. The patient’s general condition, including blood pressure, improved with treatment.

**Figure 1:**
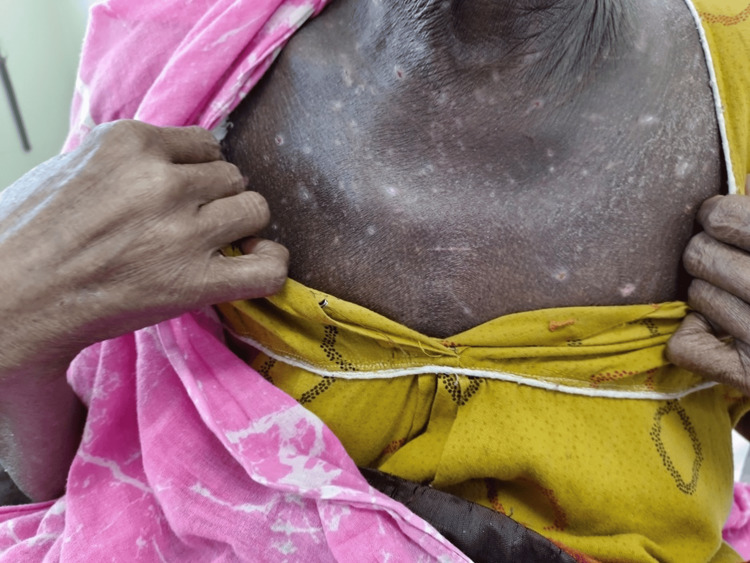
Hyperpigmented skin and hypopigmented areas over the chest

**Figure 2:**
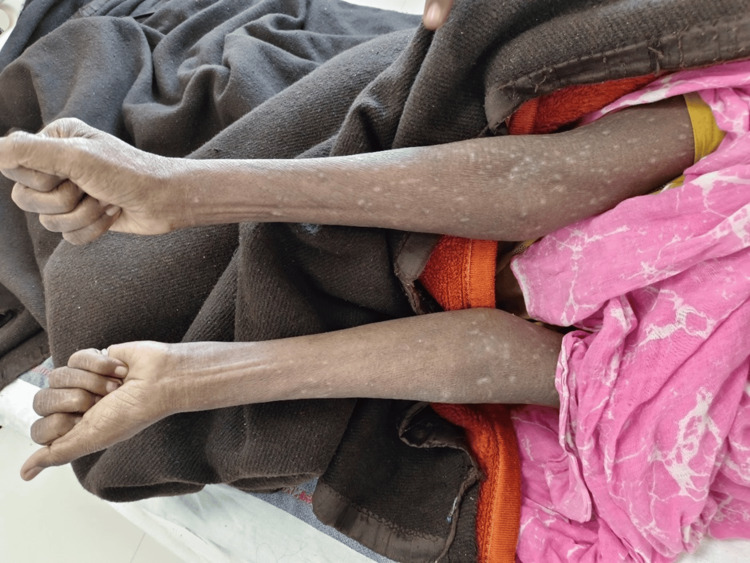
Hyperpigmented skin with few hypopigmented areas over both forearms

On follow-up, generalized itching persisted, and there was occurrence of erythematous scaly patches at the site of the itching that converted to hyperpigmented scaly areas (Figures 1, 2).

A provisional diagnosis of tinea corporis was made in consultation with the dermatology department of our hospital, and oral and topical antifungal medications were added to the treatment. Despite treatment, there were recurrent episodes of severe itching with mild fever lasting for 3-4 hours that could only be controlled by treatment with an injection of hydrocortisone and an injection of pheniramine. She was investigated again and was found to have leukocytosis (13,800 cells/mm^3^) and HE (23.4%). Hepatitis B, human immunodeficiency virus (HIV), and anti-HCV were nonreactive. Considering eosinophilia secondary to parasitic infections, treatment was upgraded with tablet Ivermectin 12 mg daily and tablet albendazole 400 mg daily for a total of three days. Tablet diethylcarbamazine 150 mg twice a day was also administered for 21 days.

On the next follow-up, itching was persistent despite treatment with antifungals and antihelminthic drugs. The stool analysis was negative for ova and cysts. Given the background of persistent HE and dermatological manifestations, a skin biopsy was planned to look for tissue infiltration by eosinophils and to rule out granulomatous pathology. The histopathological findings showed orthokeratotic hyperkeratosis and erythroderma that were inconclusive. The patient’s itching was persistent, which made her annoyed and anxious. She would even cry out loud and pull her hair out in reaction to the severe itching. There was appearance of enlarged bilateral cervical lymph nodes on general examination, while the systemic examination was normal. Ultrasound of the abdomen and pelvis was normal. Contrast-enhanced CT neck and thorax showed bilateral cervical and axillary lymph nodes with painless enlargement. A lymph node biopsy was done which showed dermatopathic lymphadenitis. To further investigate the generalized lymphadenopathy, a whole-body FDG-PET scan and upper gastrointestinal endoscopy were done but did not reveal any underlying neoplasm. Blood biochemistry (Table 1) was suggestive of a raised serum ferritin, normal serum vitamin B12 level, and raised serum IgE level. Bone marrow aspiration was done to rule out myeloproliferative disorder. It showed cellular bone marrow with trilineage hematopoiesis with a marked eosinophilic prominence (24%) that was suggestive of HES. Treatment was started with tablet Deflazacort 12 mg daily and tablet hydroxyurea 500 mg daily for two weeks, after which episodes of severe itching were reduced, but the eosinophilia persisted. The patient was discharged and was asked to have a follow-up after 15 days.

**Table 1:** Investigation table ACE: Angiotensin-converting enzyme; ESR: erythrocyte sedimentation rate; RF: rheumatoid factor; anti-CCP: anti-cyclic citrullinated peptide; CRP: C-reactive protein; ANA IFA: antinuclear antibody by immunofluorescence assay; AST: serum aspartate aminotransferase; ALT: serum alanine transaminase; INR: international normalized ratio; TSH: thyroid-stimulating hormone

Investigations	Patient’s Value	Reference Range
Serum Uric acid (mg/dl)	4.7	2.6-6
Serum Vitamin B_12_ assay (Pg/ml)	320.8	197-771
Serum Vitamin D total (ng/ml)	32.79	>=30
Serum ACE (U/L)	126	12-68
ESR (mm after 1 hour)	45	0-15
RF (IU/ml)	Negative	<14
Anti CCP (U/ml)	Negative	<7.0
CRP (mg/L)	38	<5.0
ANA IFA	<1:80	<1:80
AST (U/L)	24	0-45
ALT (U/L)	13	0-45
Total Bilirubin (mg/dl)	0.31	0.2-1.2
INR	1.1	<=1.1
D Dimer (μg/ml)	3.74	<0.5
Blood Urea (mg/dl)	31	15-39
Serum Creatinine (mg/dl)	0.7	0.6-1.3
Serum Sodium (mmol/L)	142	135
Serum Potassium (mmol/L)	4.1	3.5-5.5
Serum Calcium (in mg/dl)	7.4	8.4- 10.2
Random Blood Sugar (in mg/dl)	196	70-140
HbA1c	7.6%	<5.4%
Spot urine albumin creatinine ratio (mg/g)	10	<30
Total IgE (IU/ml)	7072.6	<158
Total IgM (gm/L)	0.59	0.5-3
Serum Ferritin (ng/ml)	>1650	13-150
Serum Iron (μg/dl)	26	33-193
Serum TSH (μIU/ml)	1.92	0.27-4.20
Serum Cortisol (nmol/L)	500	123-626

**Figure 3:**
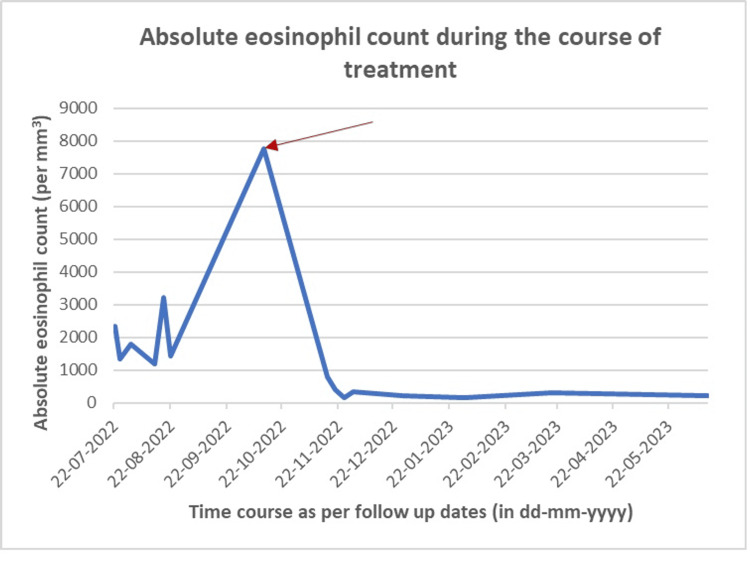
Graph showing the timeline of rise and fall in the absolute eosinophil count. The red arrow indicates the point of starting Imatinib.

On the follow-up visit, the patient still had persistent symptoms in the form of severe itching all over the body, particularly the hands and legs, with flushing and mild swelling over the face. Such episodes would recur 2-3 times per week with fever during the episodes. This was associated with darkening of the skin with hyperpigmented maculopapular areas that had gradually increased in the past two weeks. Repeated investigations showed anemia and leukocytosis with a differential leukocyte count (DLC) showing 33% eosinophils. The absolute eosinophil count was 4620/mm^3^. Considering the possibility of primary hypereosinophilic syndrome, a peripheral blood flow cytometry and next-generation sequencing for myeloid mutation gene panel were done. Meanwhile, treatment was shifted to tablet prednisolone at 1 mg/kg/day for two weeks and was tapered to 5 mg/day. Episodes of itching were still not reduced, and HE was continuously increasing up to 47%. Mutation studies for FIP1L1-CHIC2-PDGFRA, FGFR1 8p12, PDGFR B (5q32), and BCR-ABL1 qualitative mutations were negative. T cell flow cytometry by FACSCanto II showed that 15% lymphoid cells were gated using CD45, and 87% gated region T lymphoid, 1% B lymphoid, and 12% NK cells. The ratio of CD4/CD8 was 1.2. The remaining 77% were granular myeloid, and 8% were eosinophils. Treatment was started with tablet Imatinib 400 mg once daily for two weeks and tapered to 100 mg once daily for six weeks. The patient became symptomatically better, and the eosinophil counts decreased (Figure 3). She has been off imatinib and steroids for the past year. Her latest complete blood count showed normal total leukocyte count (TLC) and normal DLC with eosinophils dropped to 2.3% (as depicted in Table 2). Her absolute eosinophil count was 170/µL. The most probable diagnosis remained idiopathic HE syndrome.

**Table 2:** Trends in complete blood count at the baseline, at the rise of eosinophils, at the peak of eosinophils, and four weeks after starting treatment with tablet Imatinib. CBC: Complete blood count; TLC: total leukocyte count; AEC: absolute eosinophil count; Hb: hemoglobin.

Investigation	Baseline Values (8/6/2022)	Rise of Eosinophils (22/7/22)	Peak of Eosinophilia (26/10/2022)	After Treatment With Imatnib (28/11/2022)	Reference Values
TLC (per μL)	9.6*10^3 ^	22.56*103	16.2*10^3 ^	8.53 *10^3 ^	4-10 * 10^3 ^
Neutrophil %	73	86	37.9	77.5	4-80
Lymphocyte %	20	2.2	8.9	10.1	20-40
Monocyte %	4	1	5	7.9	2-10
Eosinophil %	3	10.3	47.9	3.3	1-6
Basophil %	0	0.1	0.3	1.2	0-1
AEC (per μL)	0.28*10^3 ^	2.33*10^3 ^	7.76 *10^3 ^	0.28 * 10^3 ^	0.02-0.5*10^3^
Hb (g/dl)	8.8	9.4	8.7	10.6	12-15
Platelet (per μL)	217*10^3 ^	526*10^3 ^	630*10^3 ^	379 *10^3^	150-450*10^3 ^

## Discussion

The classification system established by the International Cooperative Working Group on Eosinophil Disorders was developed in 2011. As per the classification, eosinophil disorders can be classified according to the following: (a) blood eosinophilia means eosinophils counts >0.5 eosinophils × 103/μL of blood; (b) hereditary (familial) HE is defined when the pathogenesis is unknown, and there is a familial clustering of cases, no signs or symptoms of hereditary immunodeficiency, and no evidence of a reactive or neoplastic condition/disorder underlying HE is found; (c) HE of undetermined significance is defined when no underlying cause of HE is found without any family history, with no evidence of a reactive or neoplastic condition/disorder underlying HE, and with no end-organ damage attributable to HE; (d) primary (clonal/neoplastic) HE is defined as an underlying stem cell, myeloid, or eosinophilic neoplasm as classified by the WHO criteria in which eosinophils are considered as neoplastic cells; and (e) secondary (reactive) HE is defined as an underlying condition/disease in which eosinophils are considered nonclonal cells; in which, the HE is mostly considered to be cytokine driven [3].

HE is identified when eosinophil levels exceed 1.5 × 103/microliter of blood in two tests conducted at least one month apart and/or when tissue HE meets any of these conditions: (a) eosinophils account for more than 20% of all nucleated cells in a bone marrow section; (b) a pathologist determines there is substantial infiltration of eosinophils in tissue; or (c) there is a significant accumulation of eosinophil granule proteins, regardless of whether major tissue infiltration by eosinophils is present or not. The HES is defined by (a) meeting the criteria for peripheral blood HE, (b) organ damage and/or dysfunction attributable to tissue HE, and (c) exclusion of other disorders or conditions as the major reason for organ damage [2]. In our case, the patient had persistent eosinophilia along with eosinophilic prominence in her bone marrow, suggestive of HES.

Reactive HE may be linked to pathologies like infections (especially those caused by parasites), allergies, autoimmune diseases, and cancers. The regulation of HE involves hematopoietic growth factors, including granulocyte-macrophage colony-stimulating factor and several interleukins (IL-2, IL-3, IL-5), with IL-5 specifically controlling eosinophil production [4]. Infections caused by helminths and certain enteric protozoa *(Isospora belli* and *Dientamoeba fragilis*) can be excluded through repeated stool and urine tests. However, infections like *Trichinella spiralis, Strongyloides stercoralis, *and *Toxocara canis*, which often lead to eosinophilia, might necessitate serological exams and tissue biopsies. In regions where filarial infections are prevalent, empirical treatment with antifilarial drugs is sometimes administered before confirming a diagnosis of HES. Moreover, the use of antiparasitic medication without a confirmed parasite infection has been suggested as a beneficial treatment for HE. Less common causes of reactive eosinophilia include dialysis, rejection of kidney transplants, IL-2 infusions, toxic oil syndrome, and infections from HIV or human lymphotropic virus-II. Other causes include the tryptophan-induced eosinophilia myalgia syndrome, Kimura’s or Weil’s disease, specific single-organ diseases associated with eosinophilia if persistent blood eosinophilia is not present (e.g., eosinophilic pneumonitis, gastroenteritis, peritonitis), patients with episodic angioedema (Gleich’s syndrome) and non-episodic angioedema, Churg-Strauss syndrome, and Omenn’s syndrome [5]. We tried to rule out all possible causes of reactive eosinophilia on the basis of clinical history, examination, investigations, and treatment approach.

Clonal eosinophilia is classified into two categories depending on whether there is a molecular rearrangement of the PDGFRA, PDGFRB, or FGFR1 genes, which are responsible for encoding the tyrosine kinase receptors that aid in eosinophil growth. The first category includes neoplasms that are either purely myeloid or both myeloid and lymphoid. The second category includes chronic eosinophilic leukemia not otherwise specified along with other conditions, such as myeloproliferative, acute myeloid, or acute biphenotypic leukemia [4]. Numerous cases have been documented where patients exhibited unexplained eosinophilia linked to a distinct group of cytokine-producing T cells in the bloodstream. These cytogenetic anomalies were limited to a group of CD3+, CD4- CD8- cells, which is the same cell phenotype observed in certain peripheral T-cell tumors [5]. In our case, flow cytometry ruled out clonal expansion, and the work-up for myeloproliferative neoplasm was also normal.

The term idiopathic HE is used to designate the clinicopathological picture when neither the diagnostic criteria for reactive HE nor those for clonal eosinophilia are met. So in our case also, it was a case of idiopathic HES. For idiopathic HE, the first-line treatment is hydroxyurea and steroids. If it is not responding, Imatinib mesylate should be considered. It is effective in medium doses at 400 mg daily [2]. In our patients, it showed a dramatic response through tyrosine kinase pathways; mutation was absent. This may be because of molecular alterations different from those that have been reported to date.

Looking at the research more broadly, it might also be beneficial to tentatively classify patients who test negative for FIP1L1-PDGFRA but show hematologic improvements with imatinib treatment as imatinib responsive. The response to imatinib observed in these individuals could suggest the presence of other clonal molecular irregularities beyond FIP1L1-PDGFRA. In such instances, it is important to explore and identify alternative targets of imatinib that play a role in the development of these disorders [6].

The current literature indicates the presence of over 50 fusion genes arising from various chromosomal and molecular irregularities. The malfunctioning activities associated with these genes underscore the crucial role of constantly active tyrosine kinases in the development of hypereosinophilic disorders. Because of resource limitations in some clinical laboratories, not all mutations linked to clonal HES can be thoroughly tested. Therefore, managing cases may focus on options related to the more common mutations. Our case emphasizes the importance of not disregarding significant treatment avenues like imatinib mesylate, which enabled us to effectively manage a severe HES condition with minimal dosage and no evident side effects. Hence, in resistant cases, considering a brief trial of therapy with imatinib mesylate after ruling out other causes of secondary eosinophilia and when there is a strong suspicion of eosinophilic clonal proliferation might be reasonable.

In our patient, we started with steroids with a partial response and later on started on imatinib, which showed a dramatic response. Our patient has been off imatinib for the past 1.5 years with a normal eosinophil count (see Figure 3).

**Figure 4:**
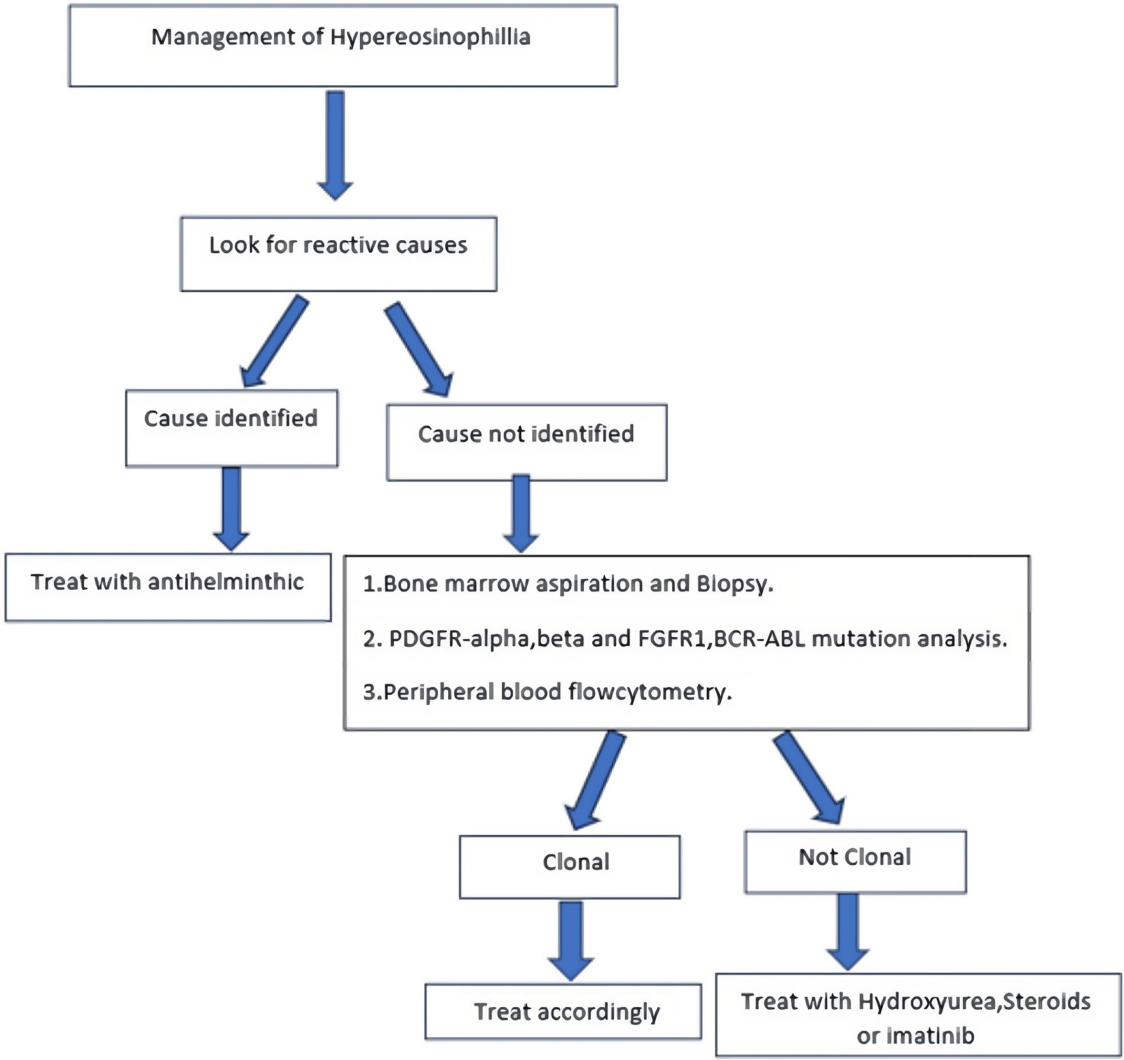
Flowchart depicting approach toward management of hypereosinophillia.

## Conclusions

Eosinophilia may be reactive, clonal, or idiopathic. In areas endemic for parasitic infections, we should first search for reactive causes and administer empirical treatment with tablet diethylcarbamazine, and other antiparasitic drugs must be considered. In idiopathic HES, treatment options are tablet prednisolone with or without tablet hydroxyurea. Imatinib is an effective second-line agent in idiopathic HE syndrome. Every patient with eosinophilia should be investigated thoroughly and require close follow-up.

## References

[1] Ogbogu PU, Bochner BS, Butterfield JH (2009). Hypereosinophilic syndrome: a multicenter, retrospective analysis of clinical characteristics and response to therapy. J Allergy Clin Immunol.

[2] Valent P, Klion AD, Horny HP (2012). Contemporary consensus proposal on criteria and classification of eosinophilic disorders and related syndromes. J Allergy Clin Immunol.

[3] Szymczyk A, Jaworski J, Podhorecka M (2024). The challenge of diagnosing and classifying eosinophilia and eosinophil disorders: a review. Cent Eur J Immunol.

[4] Hypereosinophilia Hypereosinophilia (2014). Morphology of Blood Disorders. https://doi.org/10.1002/9781118442562.ch6.

[5] Brito-Babapulle F (2003). The eosinophilias, including the idiopathic hypereosinophilic syndrome. Br J Haematol.

[6] Gotlib J, Cools J, Malone JM 3rd, Schrier SL, Gilliland DG, Coutré SE (2004). The FIP1L1-PDGFRalpha fusion tyrosine kinase in hypereosinophilic syndrome and chronic eosinophilic leukemia: implications for diagnosis, classification, and management. Blood.

